# Defucosylated Mouse–Dog Chimeric Anti-EGFR Antibody Exerts Antitumor Activities in Mouse Xenograft Models of Canine Tumors

**DOI:** 10.3390/cells10123599

**Published:** 2021-12-20

**Authors:** Guanjie Li, Tomokazu Ohishi, Mika K. Kaneko, Junko Takei, Takuya Mizuno, Manabu Kawada, Masaki Saito, Hiroyuki Suzuki, Yukinari Kato

**Affiliations:** 1 Department of Molecular Pharmacology, Tohoku University Graduate School of Medicine, 2-1 Seiryo-machi, Aoba-ku, Sendai 980-8575, Japan; clownair716@gmail.com (G.L.); saimasa@med.tohoku.ac.jp (M.S.); hiroyuki.suzuki.b4@tohoku.ac.jp (H.S.); 2 Institute of Microbial Chemistry (BIKAKEN), Numazu, Microbial Chemistry Research Foundation, 18-24 Miyamoto, Numazu-shi 410-0301, Japan; kawadam@bikaken.or.jp; 3 Department of Antibody Drug Development, Tohoku University Graduate School of Medicine, 2-1 Seiryo-machi, Aoba-ku, Sendai 980-8575, Japan; k.mika@med.tohoku.ac.jp (M.K.K.); lcdoosur@tmd.ac.jp (J.T.); 4 Laboratory of Molecular Diagnostics and Therapeutics, Joint Faculty of Veterinary Medicine, Yamaguchi University, 1677-1 Yoshida, Yamaguchi 753-8515, Japan; mizutaku@yamaguchi-u.ac.jp

**Keywords:** EGFR, mouse–dog chimeric antibody, ADCC, CDC, canine osteosarcoma, antitumor activity

## Abstract

The epidermal growth factor receptor (EGFR) contributes to tumor malignancy via gene amplification and protein overexpression. Previously, we developed an anti-human EGFR (hEGFR) monoclonal antibody, namely EMab-134, which detects hEGFR and dog EGFR (dEGFR) with high sensitivity and specificity. In this study, we produced a defucosylated mouse–dog chimeric anti-EGFR monoclonal antibody, namely E134Bf. In vitro analysis revealed that E134Bf highly exerted antibody-dependent cellular cytotoxicity and complement-dependent cytotoxicity against a canine osteosarcoma cell line (D-17) and a canine fibroblastic cell line (A-72), both of which express endogenous dEGFR. Moreover, in vivo administration of E134Bf significantly suppressed the development of D-17 and A-72 compared with the control dog IgG in mouse xenografts. These results indicate that E134Bf exerts antitumor effects against dEGFR-expressing canine cancers and could be valuable as part of an antibody treatment regimen for dogs.

## 1. Introduction

The epidermal growth factor receptor (EGFR) is a member of the receptor tyrosine kinases, which can form homo- or heterodimers with other EGFR family members, including HER2 (ErbB2/neu), HER3 (ErbB3), and HER4 (ErbB4) [1,2]. These dimers promote cell proliferation through the activation of several signaling pathways, such as the PI3K-AKT-mTOR, Ras-Raf-MEK-ERK, and JAK-STAT pathways [3]. EGFR overexpression is observed in various tumors—including lung [4], breast [5], and colorectal carcinomas [6]; glioblastomas [7]; and osteosarcomas [8,9]—and contributes to tumor malignancy by augmenting the aforementioned signaling pathways.

Osteosarcoma is the most common primary bone tumor in dogs and leads to metastasis [10]. Canine osteosarcoma has only a 45% 1-year survival rate, and its incidence is 27 times higher than that in humans [11]. The therapeutic measures for canine osteosarcoma include surgery, radiotherapy, and chemotherapy [12,13]. However, they are not sufficiently effective. Thus, new therapeutic strategies for canine osteosarcoma need to be developed.

By immunizing mice with purified recombinant hEGFR ectodomain (hEGFRec) from cultures of hEGFRec-overexpressed human glioblastoma, LN229, cells, we previously developed a novel anti-human EGFR (hEGFR) monoclonal antibody (mAb), namely EMab-134 [14]. EMab-134 can be used in Western blotting, flow cytometry, and immunohistochemistry. The mouse IgG_2a_ version of EMab-134 (134-mG_2a_) demonstrates antitumor activities in mouse xenograft models of hEGFR-expressing oral squamous cell carcinoma [15]. In addition, we produced the defucosylated version of 134-mG_2a_ (134-mG_2a_-f) to augment antibody-dependent cellular cytotoxicity (ADCC) [16]. The 134-mG_2a_-f exhibits ADCC and complement-dependent cytotoxicity (CDC) in dog EGFR (dEGFR)-overexpressed CHO-K1 (CHO/dEGFR) cells and antitumor activities in mouse xenograft models of CHO/dEGFR cells [16]. In this study, we investigated the antitumor activities of a defucosylated mouse–dog anti-EGFR mAb (E134Bf) against D-17 and A-72 xenografts, both of which endogenously express dEGFR.

## 2. Materials and Methods

### 2.1. Cell Lines

CHO-K1, a canine osteosarcoma cell line (D-17 [17]), and a canine fibroblast cell line (A-72 [18]) were obtained from the American Type Culture Collection (Manassas, VA, USA). CHO/dEGFR was established in our previous study [16]. CHO-K1 and CHO/dEGFR were cultured in RPMI medium (Nacalai Tesque, Inc., Kyoto, Japan), D-17 in Minimum Essential Medium (Nacalai Tesque, Inc., Kyoto, Japan), and A-72 in Dulbecco’s Modified Eagle Medium (DMEM; Nacalai Tesque, Inc., Kyoto, Japan). Those media were supplemented with 10% heat-inactivated fetal bovine serum (FBS; Thermo Fisher Scientific Inc., Waltham, MA, USA), 1 mM of sodium pyruvate, 100 units/mL of penicillin, 100 μg/mL of streptomycin, and 0.25 μg/mL of amphotericin B (Nacalai Tesque, Inc., Kyoto, Japan). The cells were cultured at 37 °C in a humidified atmosphere containing 5% CO_2_. The negativity for mycoplasma infection was confirmed using the TaKaRa Mycoplasma Detection Set (Takara Bio, Shiga, Japan).

### 2.2. Animals

All animal experiments were conducted according to relevant guidelines and regulations to minimize animal suffering and distress in the laboratory. Animal experiments for antitumor activity were approved by the Institutional Committee for Experiments of the Institute of Microbial Chemistry (permit No. 2021-021). The mice were maintained in a specific pathogen-free environment (23 ± 2 °C, 55 ± 5% humidity) on an 11 h light/13 h dark cycle with food and water supplied *ad libitum* throughout the experimental period. In addition, the mice were monitored for health and weight every 2–5 days during the 3-week period for each experiment. We determined the loss of original body weight of more than 25% or a maximum tumor size greater than 3000 mm^3^ as humane endpoints for euthanasia. The mice were euthanized by cervical dislocation and death was verified by respiratory and cardiac arrest.

### 2.3. Antibodies

The anti-hEGFR mAb, EMab-134, was developed as previously described [14]. To produce E134B, we subcloned the V_H_ cDNA of EMab-134 and C_H_ cDNA of dog IgGB into the pCAG-Ble vector (FUJIFILM Wako Pure Chemical Corporation, Osaka, Japan), along with the V_L_ cDNA of EMab-134 and C_L_ cDNA of dog kappa light chain into the pCAG-Neo vector (FUJIFILM Wako Pure Chemical Corporation). Two vectors of E134B were transfected into BINDS-09 cells (FUT8-deficient ExpiCHO-S cells) using the ExpiCHO Expression System (Thermo Fisher Scientific Inc., Waltham, MA, USA) [16]. The resulting mAb, E134Bf, was purified with Protein G-Sepharose (GE Healthcare Biosciences, Pittsburgh, PA, USA) [16]. Dog IgG was purchased from Jackson ImmunoResearch Inc. (West Grove, PA, USA).

### 2.4. Flow Cytometry

The D-17 and A-72 cells (1 × 10^5^ cells/sample) were harvested by brief exposure to 0.25% trypsin in 1 mM ethylenediamine tetraacetic acid (EDTA, Nacalai Tesque, Inc.). After washing with a blocking buffer of 0.1% bovine serum albumin (BSA) in phosphate-buffered saline (PBS), the cells were treated with 1 μg/mL of E134Bf or control blocking buffer for 30 min at 4 °C. The cells were then incubated in FITC-conjugated anti-dog IgG at a dilution of 1:1000 (Thermo Fisher Scientific Inc.) for 30 min at 4 °C. Fluorescence data were collected using 488 nm laser and 525/50 bandpass filter of the EC800 Cell Analyzer (Sony Corp., Tokyo, Japan). No gating was used for the data analysis. All experiments were conducted in triplicate.

### 2.5. Determination of Binding Affinity

The D-17 and A-72 cells (1 × 10^5^ cells/sample) were suspended in 100 μL of serially diluted E134Bf (0.006–100 μg/mL), followed by FITC-conjugated anti-dog IgG (1:200). Fluorescence data were collected using 488 nm laser and 525/50 bandpass filter of the EC800 Cell Analyzer. No gating was used for the data analysis. All experiments were conducted in triplicate. The dissociation constant (*K*_D_) was calculated by fitting the binding isotherms to built-in one-site binding models in GraphPad Prism 8 (GraphPad Software, Inc., La Jolla, CA, USA).

### 2.6. Immunocytochemistry

The D-17 and A-72 cells (1 × 10^5^ cells/sample) were fixed with 4% paraformaldehyde in PBS for 10 min and quenched with 50 mM NH_4_Cl in PBS containing 0.2 mM Ca^2+^ and 2 mM Mg^2+^ (PBSc/m) for 10 min. The cells were treated with blocking buffer (PBSc/m supplemented with 0.5% BSA) for 30 min and incubated with 10 µg/mL of E134Bf or the control blocking buffer for 1 h. The cells were then incubated with Alexa Fluor 488-conjugated anti-dog IgG (1:400; Jackson ImmunoResearch Laboratories, Inc., West Grove, PA, USA) and DAPI (Thermo Fisher Scientific Inc.) for 45 min. Fluorescence images were obtained using a BZ-X800 digital microscope (Keyence, Osaka, Japan) at a magnification of 40× with a GFP filter channel (green) and a DAPI filter channel (blue). All experiments were conducted in triplicate.

### 2.7. ADCC

ADCC assay was performed as previously described [16,19,20,21,22,23,24,25,26]. Briefly, canine mononuclear cells derived from dog blood were obtained from Yamaguchi University and resuspended in DMEM with 10% FBS to be used as effector cells. Target cells (D-17 and A-72) were labeled with 10 μg/mL calcein AM (Thermo Fisher Scientific, Inc.) and resuspended in the same medium. The target cells (2 × 10^4^ cells/well) were plated in 96-well plates and mixed with effector cells (effector/target cells ratio, 50:1), 100 μg/mL of E134Bf or the control dog IgG. After 4.5 h of incubation at 37 °C, the release of calcein into the supernatant was measured in each well. The fluorescence intensity was determined using a microplate reader (PowerScan HT; BioTek Instruments, Inc., Winooski, VT, USA) with excitation and emission wavelengths of 485 and 538 nm. Cytolytic activity (% lysis) was calculated as follows: % lysis = (E − S)/(M − S) × 100, where E denotes the fluorescence measured of the combined cultures of the target and effector cells; S, the spontaneous fluorescence of the target cells only; and M, the maximum fluorescence measured following the lysis of all cells with a buffer containing 0.5% Triton X-100, 10 mM Tris-HCl (pH 7.4), and 10 mM EDTA. All experiments were conducted in triplicate.

### 2.8. CDC

The CDC assay was performed as described previously [27,28,29]. Briefly, D-17 and A-72 cells were labeled with 10 μg/mL calcein AM and resuspended in the medium. They were then plated in 96-well plates at 2 × 10^4^ cells/well with rabbit complement (final dilution 1:10; Low-Tox-M Rabbit Complement; Cedarlane Laboratories, Hornby, ON, Canada) and 100 μg/mL of E134Bf or control dog IgG. After 4 h of incubation at 37 °C, we measured the release of calcein into the supernatant of each well. The fluorescence intensity was calculated as described in Section 2.7. All experiments were conducted in triplicate.

### 2.9. Influence of E134Bf on EGF-Stimulated Cell Growth

A-72 cells (1 × 10^4^ cells/well) were plated in each well of a 96-well plate. The cells were either left untreated or treated with 1 ng/mL of canine recombinant EGF (SinoBiological Inc., Beijing, China) with or without 20 μg/mL of normal dog IgG or E134Bf (*n* = 3 in each group). After 36 h of incubation, cell growth was determined using the CellTiter 96 Aqueous One Solution Cell Proliferation Assay Kit (Promega, Madison, WI, USA), according to the manufacturer’s protocol.

### 2.10. Antitumor Activity of E134Bf in Xenografts of D-17 and A-72 Cells

A total of 16 female BALB/c nude mice (5 weeks old, weighing 14–17 g) were purchased from Charles River Laboratories, Inc (Kanagawa, Japan)., and used in experiments once they reached 7 weeks old. D-17 or A-72 cells (0.3 mL of 1.33 × 10^8^ cells/mL in DMEM) were mixed with 0.5 mL BD Matrigel Matrix Growth Factor Reduced (BD Biosciences, San Jose, CA, USA); 100 μL of this suspension (5 × 10^6^ cells) was subcutaneously injected into the left flank of the mice.

On day 7 post-inoculation, 100 μg of E134Bf (*n* = 8) or control dog IgG (*n* = 8) in 100 μL PBS was intraperitoneally injected. Additional antibody inoculations were performed on days 14 and 21. This regimen was selected based on prior studies [15,30]. Canine mononuclear cells, which were obtained from Yamaguchi University, were injected surrounding the tumors on days 7, 14, and 21. At 25 days following cell implantation, all mice were euthanized by cervical dislocation. The tumor diameters and volumes were determined as previously described [16].

### 2.11. Immunohistochemistry

Immunohistochemistry was conducted as previously described [30]. Briefly, paraffin-embedded xenograft tumor tissues were sectioned and placed on glass microscope slides. After the sections were deparaffinized, they were boiled in buffered sodium citrate solution (0.01 mol/L, pH 6.0) for 10 min and subjected to immunohistochemical staining with anti-Ki-67 antibody (ab15580, 1:1000; Abcam, Cambridge, UK), followed by horseradish peroxidase-conjugated secondary antibody for rabbit IgG for 30 min. Then, the tissues were stained with 3,3′-diaminobenzidine using the ChemMate EnVision Kit (Agilent Technologies, Inc., Santa Clara, CA, USA). The slides were briefly immersed in hematoxylin for counterstaining and then observed under a Nikon Biophot microscope (Nikon, Tokyo, Japan) and photographed using a digital camera (Nikon Digital Sight DS-Ri1, Nikon). The photographs were taken under 400× magnification (Supplementary Figure S3A). Ki-67-positive cells were counted from five randomly selected fields (Supplementary Figure S3B).

### 2.12. Statistical Analyses

All data are expressed as mean ± standard error of the mean (SEM). Statistical analysis was conducted with Tukey’s test for ADCC and CDC and Welch’s *t*-test for tumor weight. ANOVA and Sidak’s multiple comparisons tests were conducted for tumor volume and mouse weight. All calculations were performed using GraphPad Prism 8. A *p*-value of <0.05 was considered statistically significant.

## 3. Results

### 3.1. Flow Cytometry Analysis against D-17 and A-17 Cells Using E134Bf

**Figure 1:**
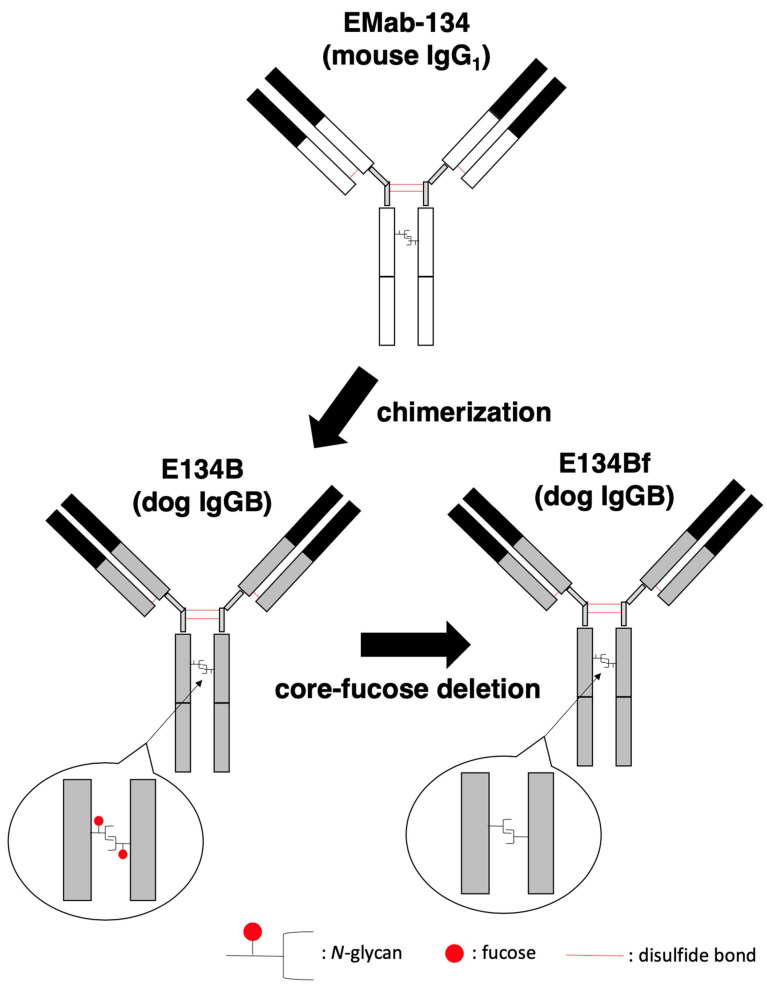
Production of E134Bf (core-fucose-deficient dog IgGB) from EMab-134 (mouse IgG_1_).

**Figure 2:**
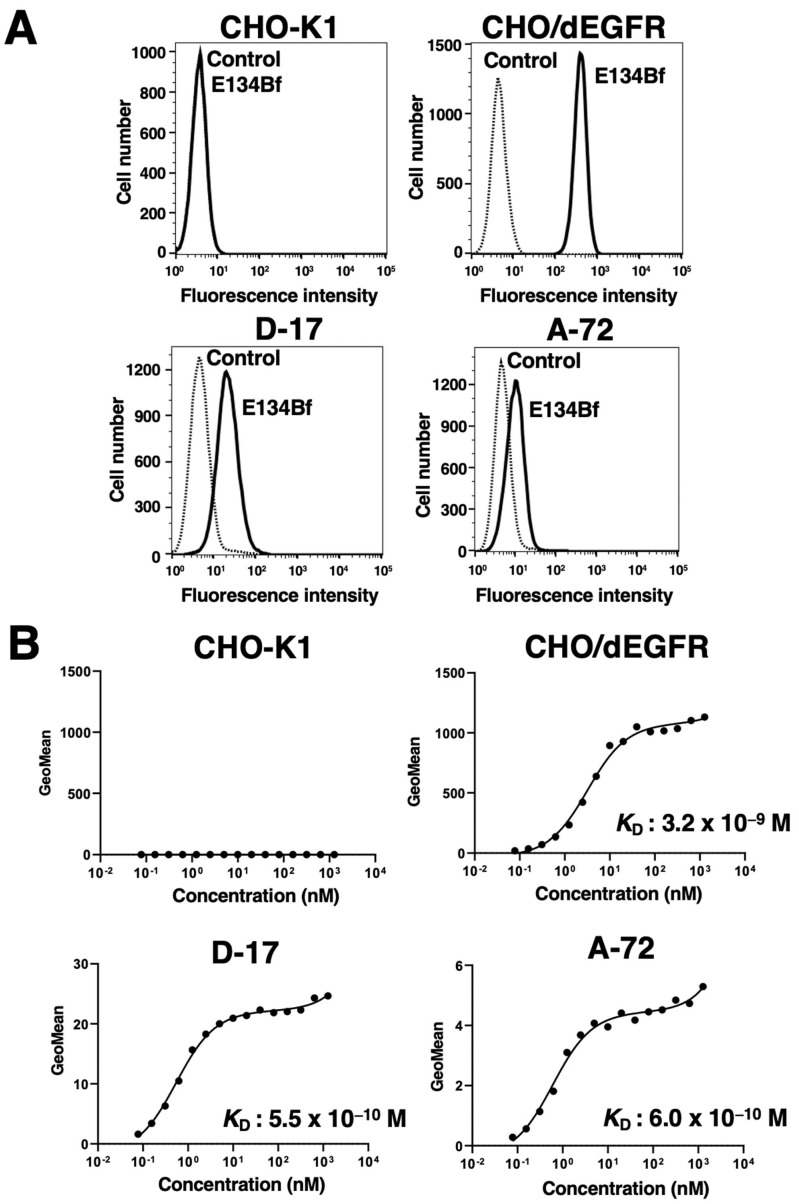
Flow cytometry using E134Bf. (**A**) CHO-K1, CHO/dEGFR, D-17, and A-72 cells were treated with E134Bf or buffer control, followed by FITC-conjugated anti-dog IgG. (**B**) Determination of the binding affinity of E134Bf for CHO-K1, CHO/dEGFR, D-17, and A-72 cells via flow cytometry. The cells were suspended in 100 μL of serially diluted E134Bf, followed by the addition of FITC-conjugated anti-dog IgG. Fluorescence data were collected using the EC800 Cell Analyzer.

In our previous study, an anti-hEGFR mAb (EMab-134) recognized dEGFR-overexpressed CHO/dEGFR cells, indicating that EMab-134 crossreacts with dEGFR [16]. In this study, we produced a defucosylated mouse–dog chimeric anti-EGFR mAb (E134Bf) by combining the V_H_ and V_L_ of EMab-134 with the C_H_ and C_L_ of dog IgG, respectively (Figure 1). E134Bf also detected canine cell lines, such as D-17 and A-72 cells, although the endogenous dEGFR expression level is lower than that of CHO/dEGFR cells (Figure 2A).

Kinetic analysis of the interactions of E134Bf with the D-17 and A-72 cells was conducted via flow cytometry. As presented in Figure 2B, the *K*_D_ for the interaction of E134Bf with the D-17 and A-72 cells was 5.5 × 10^−10^ M and 6.0 × 10^−10^ M, respectively, indicating that E134Bf exhibits high affinity for D-17 and A-72 cells. On the contrary, the *K*_D_ for the interaction between the E134Bf and CHO/dEGFR cells was 3.2 × 10^−9^ M, indicating that the binding affinity of E134Bf for endogenous dEGFR in canine cancer cells is higher than that for exogenous dEGFR in CHO/dEGFR. Since dEGFR was not detected in CHO-K1 cells, we could not determine the binding affinity of E134Bf for CHO-K1 cells.

### 3.2. Immunocytochemistry against the D-17 and A-72 Cells Using E134Bf

**Figure 3:**
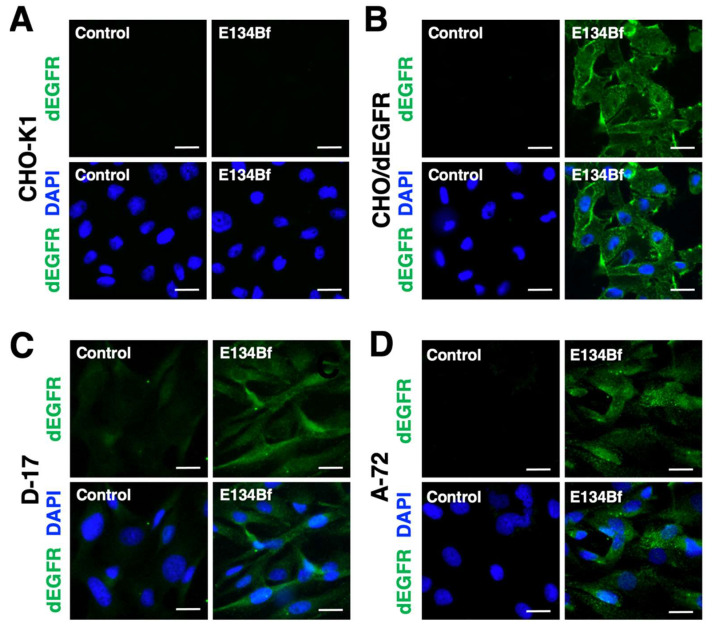
Immunocytochemistry using E134Bf. CHO-K1 (**A**), CHO/dEGFR (**B**), D-17 (**C**), and A-72 (**D**) cells were incubated with a buffer control or 10 µg/mL of E134Bf for 1 h, followed by incubation with Alexa Fluor 488-conjugated anti-dog IgG and DAPI for 45 min. Fluorescence images were taken using a fluorescence microscope BZ-X800. Scale bars, 20 µm.

Here, we investigated whether E134Bf was applicable for immunocytochemistry. At first, we evaluated the specificity of E134Bf using the CHO-K1 and CHO/dEGFR cells. As a result, E134Bf detected dEGFR on the CHO/dEGFR cells but not the CHO-K1 cells (Figure 3A,B). The buffer control detected no signal for both the CHO-K1 and CHO/dEGFR cells. Next, we examined the binding of E134Bf to endogenous dEGFR on the D-17 and A-72 cells and found that E134Bf specifically detected endogenous dEGFR (Figure 3C,D). These results indicate that E134Bf recognizes endogenous and exogenous dEGFR in immunocytochemistry.

### 3.3. E134Bf-Mediated ADCC and CDC in the D-17 and A-72 Cells

**Figure 4:**
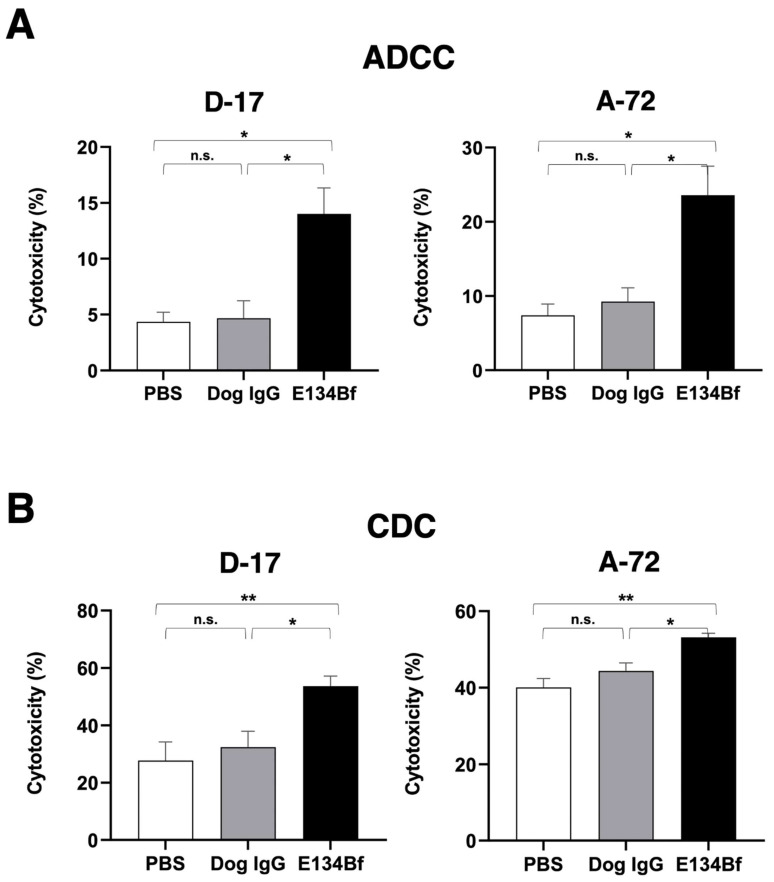
Evaluation of ADCC and CDC elicited by E134Bf. (**A**) ADCC elicited by E134Bf, control dog IgG, or control PBS targeting the D-17 and A-72 cells. (**B**) CDC elicited by E134Bf, control dog IgG, or control PBS targeting the D-17 and A-72 cells. The values are expressed as mean ± SEM. Asterisks indicate statistical significance (** *p* < 0.01; * *p* < 0.05; n.s., not significant; Tukey’s *post hoc* test). ADCC, antibody-dependent cellular cytotoxicity; CDC, complement-dependent cytotoxicity.

We investigated whether E134Bf was capable of mediating ADCC against the D-17 and A-72 cells. As presented in Figure 4A, E134Bf showed ADCC (14.0% cytotoxicity) against D-17 cells more effectively than did the control dog IgG (4.7% cytotoxicity; *p* < 0.05) and the control PBS (4.3% cytotoxicity; *p* < 0.05). E134Bf also showed ADCC (23.6% cytotoxicity) against A-72 cells more effectively than did the control dog IgG (9.2% cytotoxicity; *p* < 0.05) and the control PBS (7.4% cytotoxicity; *p* < 0.05). On the contrary, E134Bf did not show ADCC against CHO-K1 cells, which are EGFR-negative cells (Supplementary Figure S1A).

We then investigated whether E134Bf could mediate CDC against D-17 cells. As presented in Figure 4B, E134Bf elicited a higher degree of CDC (53.6% cytotoxicity) in D-17 cells compared with that elicited by the control dog IgG (32.4% cytotoxicity; *p* < 0.05) and the control PBS (27.7% cytotoxicity; *p* < 0.01). E134Bf also elicited a higher degree of CDC (53.2% cytotoxicity) in the A-72 cells compared with that elicited by control dog IgG (44.4% cytotoxicity; *p* < 0.05) and the control PBS (40.0% cytotoxicity; *p* < 0.01). On the contrary, E134Bf did not show CDC against CHO-K1 (Supplementary Figure S1B). These results indicated that E134Bf promoted significantly higher levels of ADCC and CDC against the dEGFR-expressing D-17 and A-72 cells.

### 3.4. E134Bf Did Not Inhibit the EGF-Stimulated Cell Growth of A-72

We next determined whether E134Bf could inhibit the EGF-stimulated EGFR activation, which leads to cell growth. As presented in Supplementary Figure S2, we identified A-72 as an EGF-responsive cell line. Although the addition of EGF resulted in cell growth and survival of A-72, E134Bf did not inhibit the cell growth of A-72, similar to the control dog IgG. These results indicated that E134Bf did not inhibit the activation of EGFR in response to EGF stimulation.

### 3.5. Antitumor Activities of E134Bf in the Mouse Xenografts of D-17 and A-72 Cells

**Figure 5:**
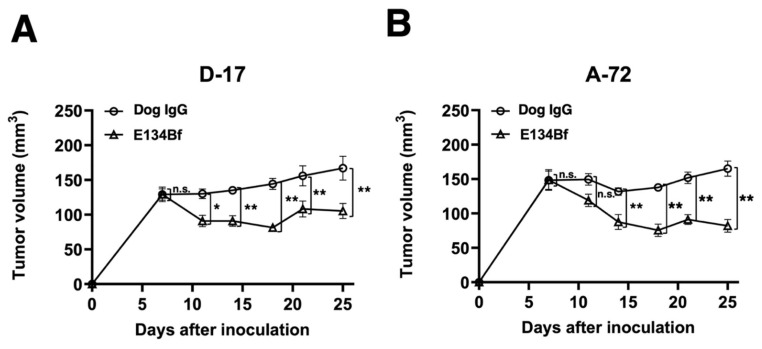
Evaluation of tumor volume in the D-17 and A-72 xenograft models. (**A**) D-17 cells (5 × 10^6^ cells) were subcutaneously injected into the left flank. On day 7, 100 μg of E134Bf (*n* = 8) or control dog IgG (*n* = 8) in 100 μL PBS was intraperitoneally injected into the mice; additional antibodies were then injected on days 14 and 21. The tumor volume was measured on days 7, 11, 14, 18, 21, and 25 after the injection. (**B**) The A-72 cells (5 × 10^6^ cells) were subcutaneously injected into the left flank. On day 7, 100 μg of E134Bf (*n* = 8) or control dog IgG (*n* = 8) in 100 μL PBS was intraperitoneally injected into the mice; additional antibodies were then injected on days 14 and 21. The tumor volume was measured on days 7, 11, 14, 18, 21, and 25 after the injection. The values are expressed as mean ± SEM. Asterisks indicate statistical significance (** *p* < 0.01; * *p* < 0.05; n.s., not significant; ANOVA and Sidak’s multiple comparisons test).

In the D-17 xenograft models, E134Bf (100 μg) and dog IgG (100 μg) were injected intraperitoneally into mice on days 7, 14, and 21, following the injection of the D-17 cells. The tumor volume was measured on days 7, 11, 14, 18, 21, and 25 post-injection. The administration of E134Bf resulted in a significant reduction in tumor development on days 11 (*p* < 0.05), 14 (*p* < 0.01), 18 (*p* < 0.01), 21 (*p* < 0.01), and 25 (*p* < 0.01) compared with that of the dog IgG (Figure 5A). The administration of E134Bf resulted in a 37% reduction in tumor volume compared with that of the control dog IgG on day 25 post-injection.

In the A-72 xenograft models, the administration of the antibody treatment and monitoring of the tumor volume were performed the same with the D-17 cells. The administration of E134Bf resulted in a significant reduction in tumor development on days 14 (*p* < 0.01), 18 (*p* < 0.01), 21 (*p* < 0.01), and 25 (*p* < 0.01) compared with that of the dog IgG (Figure 5B). The administration of E134Bf resulted in a 50% reduction in tumor volume compared with that of the dog IgG on day 25 post-injection.

**Figure 6:**
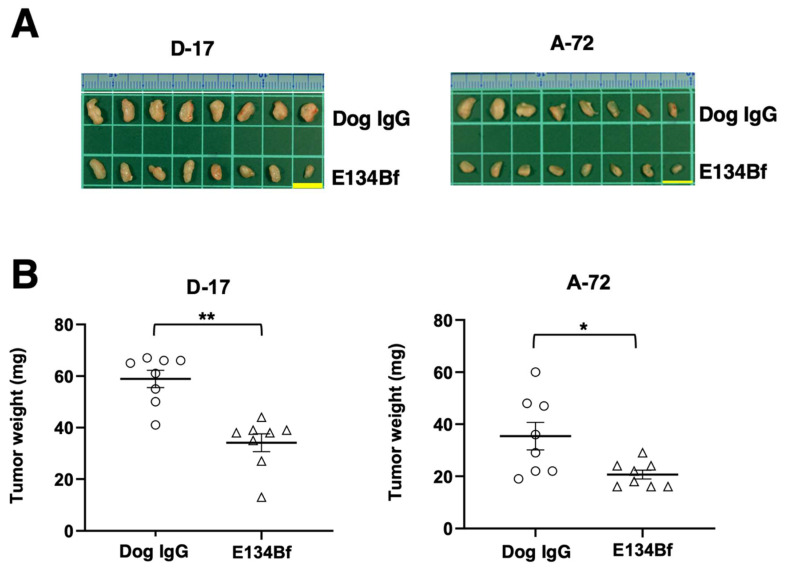
Evaluation of tumor weight in the D-17 and A-72 xenograft models. The tumors of the D-17 and A-72 xenografts were resected from the control dog IgG and E134Bf groups. (**A**) Appearance of resected tumors of the D-17 and A-72 xenografts from the control dog IgG and E134Bf groups on day 25. Scale bar, 1 cm. (**B**) The tumor weight on day 25 was measured from the excised D-17 and A-72 xenografts. The values are expressed as mean ± SEM. Asterisk indicates statistical significance (** *p* < 0.01, * *p* < 0.05, Welch’s *t*-test).

The tumors of D-17 that were resected from mice on day 25 are presented in Figure 6A. The D-17 tumors from the E134Bf-treated mice weighed significantly less than those from the control dog IgG-treated mice (42% reduction; *p* < 0.01, Figure 6B). Consistent with these results, Ki-67 staining revealed a markedly reduced proliferation index in E134Bf-treated tumors (Supplementary Figure S3). The A-72 tumors that were resected from mice on day 25 are demonstrated in Figure 6A. The A-72 tumors from the E134Bf-treated mice weighed significantly less than those from the dog IgG-treated mice (42% reduction; *p* < 0.05, Figure 6B).

**Figure 7:**
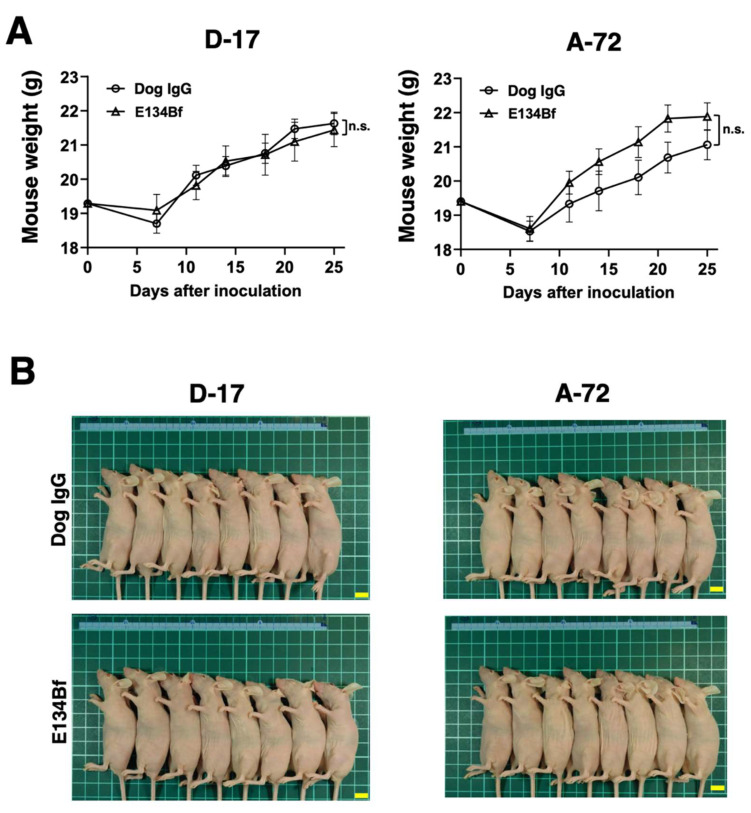
Body weights and appearance of the mice implanted with the D-17 and A-72 xenografts. (**A**) Body weights of mice implanted with the D-17 and A-72 xenografts were recorded on days 7, 11, 14, 18, 21, and 25 (n.s.: not significant). (**B**) Body appearance of the D-17 and A-72 xenograft mice on day 25. Scale bar, 1 cm. n.s., not significant.

The total body weights of the D-17 xenograft mice did not significantly differ among the two groups (Figure 7A). The body appearance of the D-17 xenograft mice on day 25 is presented in Figure 7B. In the same way, the total body weights of the A-72 xenograft mice did not significantly differ among the two groups (Figure 7A). The body appearance of the A-72 xenograft mice on day 25 is demonstrated in Figure 7B.

Taken together, these results indicate that the administration of E134Bf effectively reduced the growth of D-17 and A-72 xenografts.

## 4. Discussion

Among canine cancers, osteosarcoma is a highly metastatic and intractable cancer, and about 80% of dogs with osteosarcoma die from lung metastases [10,31,32]. Because canine osteosarcoma shares various molecular and clinical similarities with human osteosarcoma, canine osteosarcoma can be used for identifying biomarkers and developing treatments for human osteosarcoma [11]. Therefore, the development of therapeutic strategies for canine osteosarcoma will also improve the clinical response rate of human osteosarcoma. In canine osteosarcoma, surgery is a first-line treatment. Chemotherapy, including carboplatin, cisplatin, and doxorubicin, is also used for adjuvant and/or neoadjuvant therapy [33,34,35,36]. These treatments have been shown to result in longer survival times than amputation alone [37]. Because the toxicity of chemotherapy often causes severe adverse effects, such as vomiting, diarrhea, anorexia, and myelosuppression, which lead to a significant reduction in the canine’s quality of life, it is important to establish a new therapeutic modality.

Canine fibrosarcoma is a malignant, infiltrating, mesenchymal tumor that mainly affects the oral cavity of medium to large dogs [38]. In addition to surgery and radiotherapy, chemotherapy has been used as adjuvant treatment for oral fibrosarcoma. Doxorubicin is the most commonly administered drug in association with surgery and/or radiation. However, the ability to control local disease still represents the major challenge. Recently, the effect of two tyrosine kinase inhibitors (TKI), imatinib (BCR-ABL inhibitor) and masitinib (c-KIT and PDGF inhibitor), on canine fibrosarcoma cells was investigated [39]. However, there are no reports on the use of anti-EGFR drugs, including TKI or antibody drugs, for the treatment of canine oral fibrosarcoma.

Antibody therapies are successful against various diseases and generally well tolerated in humans [40]. Original antibody therapies for dogs have not been established for most diseases, including osteosarcoma; therefore, antibody drugs for humans have been used as alternatives. For example, several tumor antigens that have been used for targeted therapies in human cancers are also identified in canine malignancies, including EGFR, HER2, VEGFR2, CD20, podoplanin, PD-1, and PD-L1 [16,41,42,43,44,45,46,47,48,49]. Particularly, the hEGFR and dEGFR amino acid sequences are 91% identical, and some anti-hEGFR mAbs are effective against canine tumors that overexpress dEGFR in vitro and/or in vivo [16,50].

Although it has been reported that the aberrant expression of EGFR in human osteosarcoma is associated with poor response to chemotherapy, distant metastasis, and reduced survival time of patients [51,52], precise information on EGFR expression and its association with clinical features in canine osteosarcoma has not been elucidated. In addition, the prognostic and clinicopathological significance of the EGFR expression of canine osteosarcoma and its relevance to EGFR-targeted drugs have not been fully elucidated. Further exploring these relationships could provide new insights into the efficacy of EGFR-targeted therapies for both humans and dogs.

D-17 is a canine osteosarcoma cell line commonly used for various studies [53,54,55,56,57]. Since the D-17 cells express EGFR [58,59], Mantovani et al. applied an EGFR tyrosine kinase inhibitor to suppress D-17 cell proliferation [58]. On the contrary, there has been no study on the application of an anti-EGFR mAb to EGFR-expressing canine osteosarcoma cell lines. In this study, we demonstrated that E134Bf could recognize the endogenous dEGFR of D-17 cells via flow cytometry and immunocytochemistry (Figure 2A and Figure 3). The most critical aim of the present study was to investigate the antitumor activity of E134Bf for endogenous dEGFR-expressing canine tumor cells. E134Bf demonstrated growth inhibition of endogenous dEGFR-expressing D-17 cells without body weight loss and skin abnormality (Figure 5, Figure 6 and Figure 7).

A canine fibroblast cell line, A-72, was established from a tumor surgically removed from a golden retriever and mainly used for virus research [18]. Although the histology of the original tumor has not been identified, the A-72 cells expressed EGFR (Figure 2 and Figure 3), and its growth was promoted by EGF (Supplementary Figure S2). Furthermore, E134Bf could suppress xenograft growth (Figure 5 and Figure 6), most likely through ADCC activity (Figure 4). Furthermore, through immunocytochemistry (Figure 3), the cytosolic dot-like staining of dEGFR was preferentially observed in the A-72 cells, suggesting that the internalized dEGFR abundantly accumulates in the cytoplasm. Previously, we developed the mouse–canine chimeric anti-dog podoplanin mAb P38B, conjugated with emtansine as the payload, which demonstrated an antitumor effect against dog podoplanin -overexpressed cells [60]. It would be interesting to examine the sensitivity of an E134Bf-drug conjugate to the A-72 cells.

These results suggest that E134Bf may be useful for an antibody treatment regimen for dEGFR-expressing canine tumors, which would lead to the establishment of EGFR-targeted immunotherapy. However, this study was limited by the number of canine tumor samples. Future investigations are needed to test the antitumor effects of E134Bf against spontaneously developed canine tumors and its efficacy compared to other caninized monoclonal antibodies.

## Data Availability

The datasets used and/or analyzed during the study are available from the corresponding author on reasonable request.

## Supplementary Materials

The following are available online at https://www.mdpi.com/article/10.3390/cells10123599/s1. Figure S1: Evaluation of ADCC and CDC by E134Bf against CHO-K1, EGFR-negative cells elicited., Figure S2: E134Bf did not inhibit the cell growth of A-72 stimulated by EGF., Figure S3: Ki-67 staining in the xenograft tumor tissues.
