# Supplementary material for: Defucosylated Mouse–Dog Chimeric Anti-EGFR Antibody Exerts Antitumor Activities in Mouse Xenograft Models of Canine Tumors
Source: Cells. 2021 Dec 20;10(12):3599. doi: 10.3390/cells10123599 (PMC8700185; doi:10.3390/cells10123599)
Supplement: Supplementary file 1 [file cells-10-03599-s001.zip › cells-1516403-supplementary.pdf]

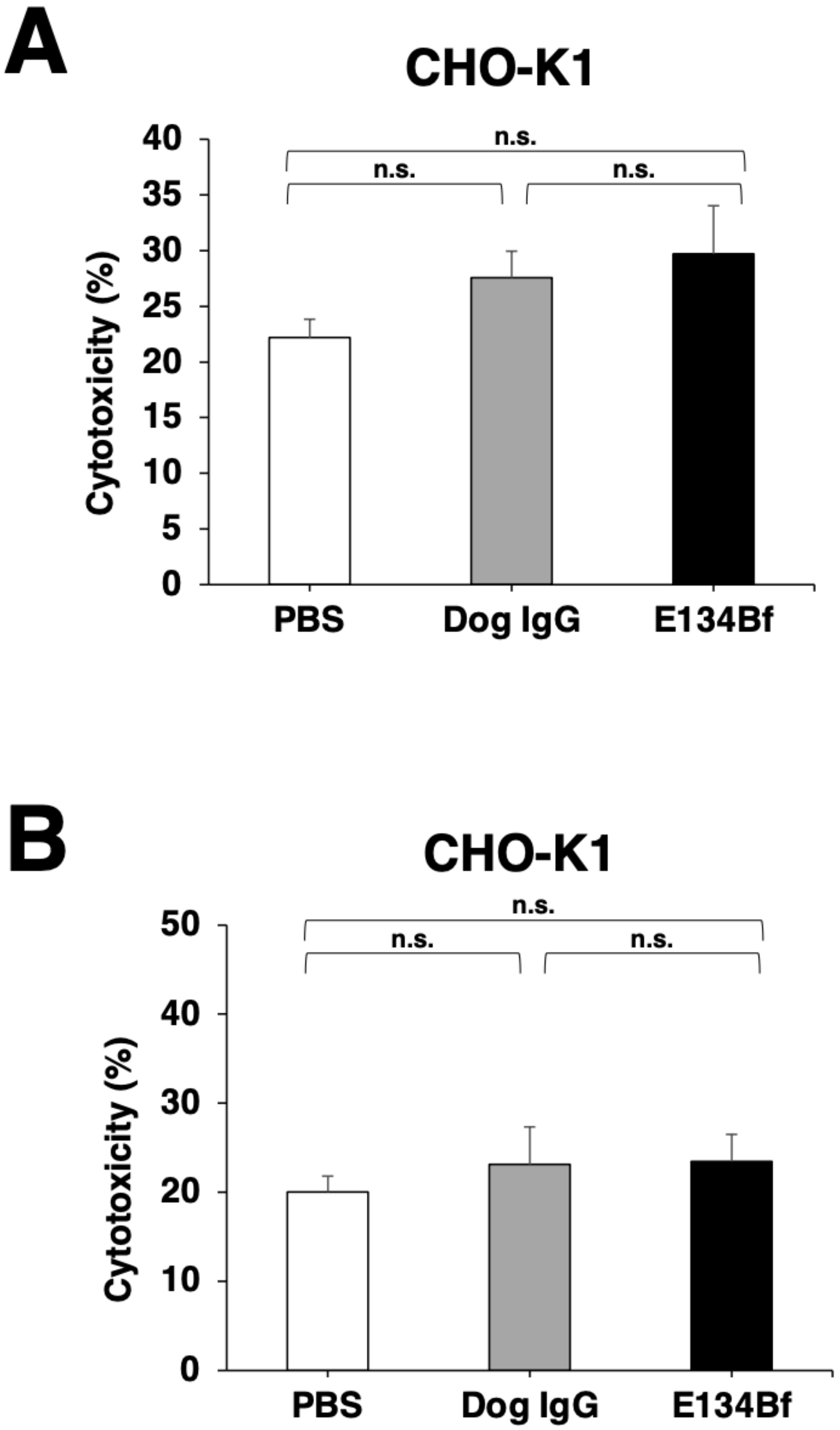

Supplementary Figure S1. Evaluation of ADCC and CDC by E134Bf against CHO-K1, EGFR-negative cells elicited. (A) ADCC elicited by E134Bf, control dog IgG, or control PBS targeting CHO-K1 cells. (B) CDC elicited by E134Bf, control dog IgG, or control PBS targeting CHO-K1 cells. The values are expressed as mean ± SEM. Asterisks indicate statistical

significance (n.s., not significant; Tukey's *post hoc* test). ADCC, antibody-dependent cellular cytotoxicity; CDC, complement-dependent cytotoxicity.

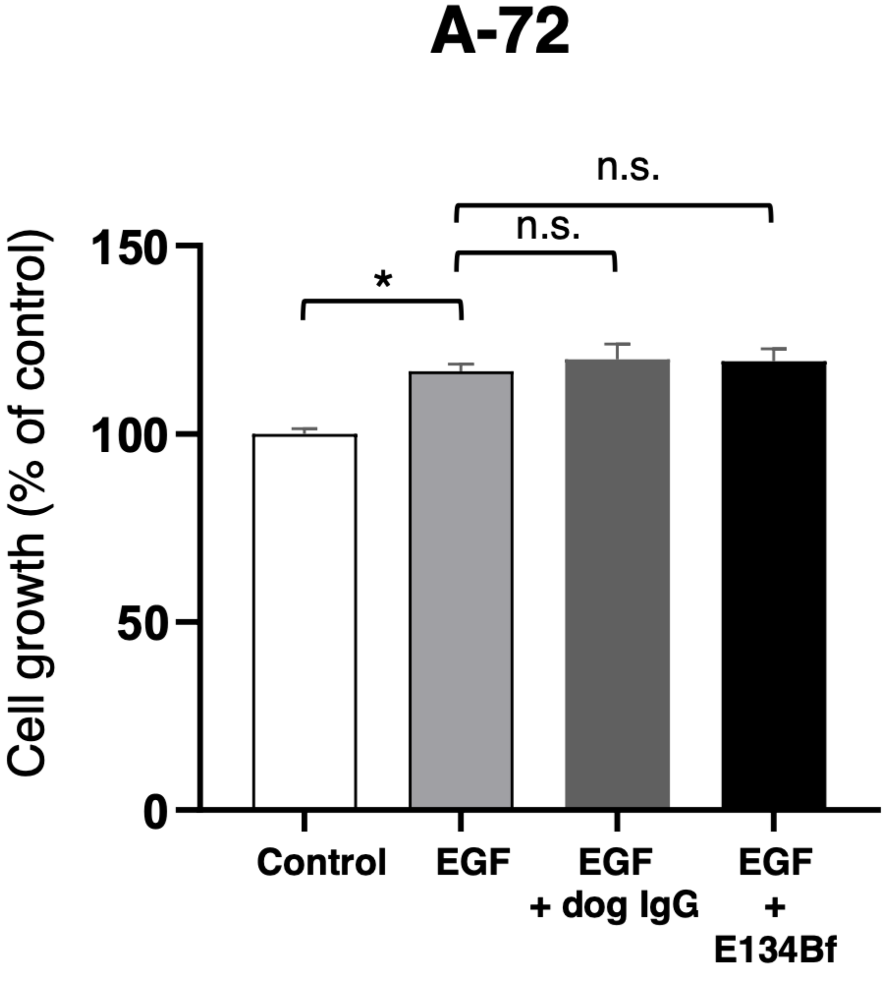

Supplementary Figure S2. E134Bf did not inhibit the cell growth of A-72 stimulated by EGF. A-72 cells were treated for 36 h with or without 1 ng/mL canine EGF or canine EGF + indicated IgGs (20 μg/mL). Cell growth was determined

using the MTS assay. The values are expressed as mean ± SEM. Asterisks indicate statistical significance (*$P < 0.05$, n.s., not significant; Tukey's *post hoc* test).

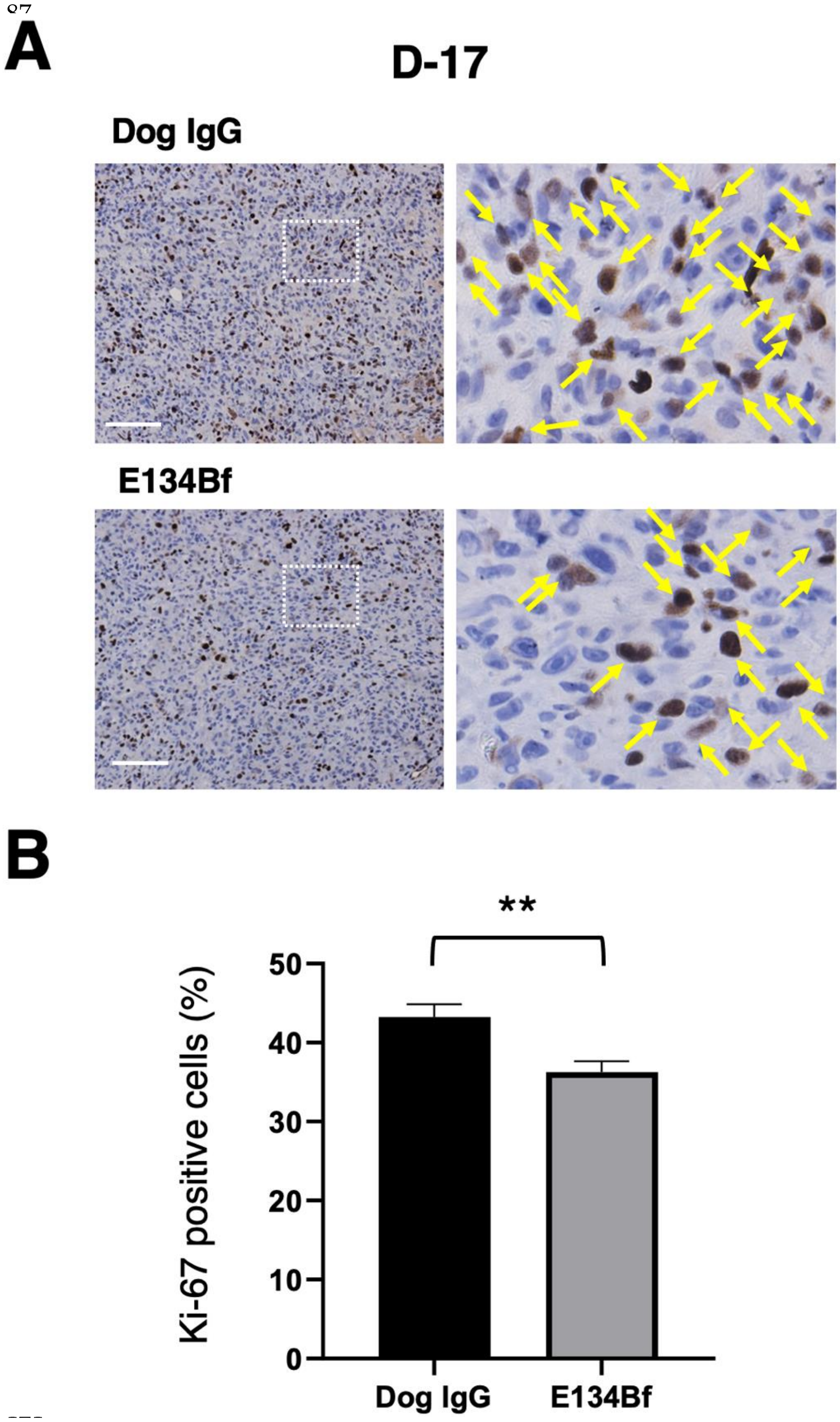

Supplementary Figure S3. Ki-67 staining in the xenograft tumor tissues. (A) The expression of Ki-67 in the tumor tissue was detected *via* immunohistochemistry. The arrows indicate Ki-67-positive cells. Scale bar, 100 μm. (B) Quantification

of Ki-67-positive cells. Five random fields were selected from within each section. The values are expressed as mean ± SEM. Asterisk indicates statistical significance (** $P < 0.01$, Welch's test).
